# Does deprivation affect breast cancer management?

**DOI:** 10.1038/sj.bjc.6602390

**Published:** 2005-02-08

**Authors:** N C Henley, D J Hole, E Kesson, H J G Burns, W D George, T G Cooke

**Affiliations:** 1 University Department of Surgery, Glasgow Royal Infirmary, Glasgow G31 2ER, UK; 2 West of Scotland Cancer Surveillance Unit, Department of Public Health, University of Glasgow, Glasgow G12 8RZ, UK; 3 Greater Glasgow National Health Service Board, 350 St Vincent Street, Glasgow G3 8YZ, UK; 4 University Department of Surgery, Western Infirmary, Glasgow G11 6NT, UK

**Keywords:** social deprivation, breast carcinoma, surgical decision-making

## Abstract

We evaluated whether social deprivation affected decision-making for breast cancer surgery. Of 3419 patients, 53.6% had mastectomy and this was predicted by deprivation, age, tumour size and hospital, all of which retained significance on multivariate analysis, except deprivation. Pathological characteristics and surgical decision-making determined choice of operation not deprivation.

Awareness of health inequalities between rich and poor has never been greater. The recent publication of health and life expectancy data in Scotland has shown that the gap between rich and poor in Glasgow persists (Clark *et al*, 2004). These inequalities are also seen in women with breast cancer. Survival differences between women from affluent areas and women from deprived areas are around 6% in England and Wales (Coleman *et al*, 2004) and 10% in Scotland (Thomson *et al*, 2001). Part of this difference is explained by more oestrogen receptor (ER) negative tumours in deprived women, but no other pathological differences have been observed (Thomson *et al*, 2001). Several other studies have looked at other pathological criteria as the reason for the persistent survival differences and they have all failed to demonstrate an association (Carnon *et al*, 1994; Macleod *et al*, 2000a; Brewster *et al*, 2001). Access to health services does not appear to be a factor; in fact, deprived women appear to use primary care resources more often than the more affluent (Macleod *et al*, 2000b). Despite this, the question remains whether deprived women are treated differently in secondary care.

Trials have shown no survival advantage from mastectomy over breast conservation surgery for tumours up to 5 cm (Fisher *et al*, 2002). The contraindications to conservation are well documented: multifocal tumours; 1st or 2nd trimester of pregnancy; history of previous irradiation to the affected breast; or a large tumour in a small breast that would result in an unacceptable cosmetic result.

We have analysed data from the Glasgow Breast Cancer Audit to measure the mastectomy rate. We hypothesised that if the mastectomy rate was higher than expected, this might be a reflection of high levels of deprivation in Glasgow (McLoone, 2004). Additionally, if surgeons were influencing women in choice of surgical management, were they actively suggesting conservation for affluent women?

## PARTICIPANTS AND METHODS

The Breast Cancer Audit in Glasgow was set up in 1995. It prospectively collects demographic, surgical and pathological data of women with primary operable breast cancer from the five hospitals in the area staffed by specialist surgical teams.

We analysed patients diagnosed between 1996 and 2001, during which time 3541 patients were diagnosed with primary operable invasive breast cancer. Patients with tumours ⩾5 cm or locally advanced disease unsuitable for conservation were excluded. Data on tumour pathology, surgical management and patient demographics including deprivation category were collected.

Deprivation was determined using the method of Carstairs and Morris (McLoone, 2004). Postcode sectors are analysed for the prevalence of various census variables associated with socioeconomic status, these are: ownership of a car, proportion of people in social classes IV and V, overcrowding and male unemployment. Postcode sectors are scored and categorised into seven deprivation categories. Categories 1 and 2 were combined to ‘affluent’; 3, 4 and 5 were combined to ‘intermediate’; and 6 and 7 were combined to ‘deprived’. Surgical management was divided into ‘conservation surgery’ (lumpectomy with axillary staging) and ‘mastectomy’ (mastectomy with axillary staging).

Age, deprivation, tumour size, nodal status, histological grade, oestrogen receptor (ER) status and hospital were individually examined for their association with surgical management using *χ*^2^ tests of association, and then subjected to multivariate analysis.

## RESULTS

Of the 3570 patients entered onto the database, 3419 had tumours smaller than 5 cm. Of these, 1588 (46.4%) underwent conservation surgery and 1831 (53.6%) mastectomy.

On univariate analysis, deprivation, tumour size, nodal status, grade, method of diagnosis and hospital varied significantly with type of surgery (Table 1 ).

Women from deprived areas were significantly more likely to have larger, symptomatic tumours (Table 2 ). There was no significant association between deprivation and nodal status, ER status or grade (Table 2).

Stepwise logistic regression modelling showed that deprivation maintained its significance when age and year of surgery were added into the model (OR=1.118; *P*=0.015), but lost its significance when tumour size was added (OR=1.07; *P*=0.245). The multivariate analysis showed that tumour size, nodal status, histological grade, method of diagnosis and hospital were independently predictive of surgical management (Table 3 ).

## DISCUSSION

Our data show that the mastectomy rate in Glasgow is higher than reported elsewhere (Morrow *et al*, 2001). Based on figures from the United States, it has been estimated that 10% of tumours smaller than 2 cm and 30% of tumours between 2 and 5 cm require mastectomy due to a medical contraindication (Morrow *et al*, 2001). In our study, the percentages having a mastectomy were 38 and 72%, respectively. Our database does not identify which patients have a medical contraindication to conservation surgery, nor does it record the decision-making process for each patient. However, access to radiotherapy is equal for all patients and it is unlikely that a high incidence of medical contraindications would explain our relatively high mastectomy rate.

We found that women from deprived areas are more likely to have a mastectomy than women from more affluent areas. However, women from deprived areas have larger and symptomatic tumours. The uptake of breast screening in Glasgow is 68.1% (data from Scottish Breast Screening Programme) with the lowest uptake in the most deprived groups, while the UK average is 75.5% (data from NHS Breast Screening Programme). Both tumour size and method of diagnosis were independently predictive of mastectomy, so it is likely that tumour size and fewer screen-detected tumours determine surgical management rather than the biased treatment of deprived women. Therefore, to some extent, our mastectomy rate reflects higher levels of deprivation in Glasgow.

The populations served by the different hospitals are similar in age and access to radiotherapy services, although their levels of deprivation differ. In the univariate model, the relatively low mastectomy rate in hospitals 1 and 4 is due to their large breast screening practice. However, in the multivariate model, which included method of diagnosis, hospital of treatment was independently predictive of surgical management. This indicates that individual surgeons have an influence over choice of surgical management. Although guidelines have been produced recommending suitability for conservation surgery, there still appears to be a lack of consensus among surgeons.

It does appear that women from deprived areas are being treated appropriately and the choice of surgery is based on tumour characteristics. However, the wide variation in mastectomy rate between hospitals suggests a lack of consensus on the best surgical treatment of breast cancer.

## Figures and Tables

**Table 1:** Univariate analysis of factors determining surgical management

**Variable**	**Conservation (%), *N*=1588 (46.4)^a^**	**Mastectomy (%), *N*=1831 (53.6)^b^**	***χ* ^2^**	***P*-value**
*Deprivation*				
Affluent	285 (46.3)	330 (53.7)	17.301	<0.0001
Intermediate	824 (49.7)	833 (50.3)		
Deprived	479 (41.8)	668 (58.2)		
				
*Tumour size (mm)*			472.492	<0.0001
<10	380 (73.4)	138 (26.6)		
10–19	770 (57.2)	577 (42.8)		
20–29	330 (35.0)	614 (65.0)		
30–39	90 (20.4)	351 (79.6)		
40–49	17 (10.1)	151 (89.9)		
				
*Nodal status*				
Negative	1117 (56.2)	869 (43.8)	252.172	<0.0001
Positive	390 (29.6)	927 (70.4)		
Not known	81	35		
				
*Method of diagnosis*				
Screen detected	824 (66.8)	409 (33.2)	327.684	<0.0001
Symptomatic	737 (34.6)	1396 (65.4)		
Not known	26	26		
				
*Grade*				
I	507 (65.5)	267 (34.5)	158.889	<0.0001
II	667 (44.0)	848 (56.0)		
III	402 (36.6)	695 (63.4)		
Not known	12	21		
				
*Hospital*				
1	722 (52.4)	656 (47.6)	65.751	<0.0001
2	198 (36.7)	341 (63.3)		
3	95 (32.6)	196 (67.4)		
4	472 (48.6)	500 (51.4)		
5	101 (42.3)	138 (57.7)		

a Defined as lumpectomy with axillary staging.

b Defined as mastectomy with axillary staging.

**Table 2:** Univariate analysis of association between deprivation and tumour characteristics

**Variable**	**Affluent (%), *N*=615 (18.0)**	**Intermediate (%), *N*=1657 (48.5)**	**Deprived (%), *N*=1147 (33.5)**	***χ* ^2^**	***P*-value**
*Tumour size*					
<10	101 (16.4)	279 (16.8)	138 (12.0)	31.699	<0.0001
10–19	250 (40.7)	673 (40.6)	425 (37.1)		
20–29	160 (26.0)	448 (27.1)	336 (29.3)		
30–39	77 (12.5)	191 (11.5)	173 (15.1)		
40–49	27 (4.4)	66 (4.0)	75 (6.5)		
					
*ER status*					
Positive	487 (79.2)	1262 (76.2)	846 (73.8)	8.405	=0.078
Negative	112 (18.2)	337 (20.3)	267 (23.3)		
Not known	16	58	34		
					
*Nodal status*					
Negative	364 (59.2)	988 (59.6)	634 (55.3)	8.484	=0.075
Positive	224 (36.4)	619 (37.4)	474 (41.3)		
Not known	27	50	39		
					
*Method of diagnosis*					
Screen detected	204 (33.2)	700 (42.2)	331 (28.9)	55.476	<0.0001
Symptomatic	411 (66.8)	957 (57.8)	816 (71.1)		
					
*Grade*					
I	140 (22.8)	380 (23.0)	254 (22.1)	5.051	=0.282
II	245 (44.9)	755 (45.6)	485 (42.3)		
III	191 (31.2)	510 (30.8)	396 (34.5)		
Not known	7	10	12		

ER=oestrogen receptor.

**Table 3:** Multivariate analysis of factors determining surgical management

**Variable**	**Relative risk of mastectomy (95% CI)^a^**	***P*-value**
Deprivation		
Affluent	1	=0.189
Intermediate	0.93 (0.752–1.161)	
Deprived	1.102 (0.873–1.392)	
		
*Tumour size (mm)*		
<10	1	<0.0001
10–19	1.302 (1.019–1.663)	
20–29	2.330 (1.774–3.060)	
30–39	4.216 (2.982–5.960)	
40–49	10.025 (5.643–17.812)	
		
*Nodal status*		
Negative	1	<0.0001
Positive	1.950 (1.649–2.305)	
		
*Method of diagnosis*		
Screen detected	1	<0.0001
Symptomatic	2.178 (1.776–2.673)	
		
*Grade*		
I	1	<0.0001
II	1.538 (1.254–1.887)	
III	1.646 (1.314–2.061)	
		
		
*Hospital*		
1	1	<0.0001
2	1.110 (0.871–1.414)	
3	1.326 (0.977–1.799)	
4	1.353 (1.118–1.637)	
5	0.639 (0.462–0.884)	

CI=confidence interval.

a Derived from multiple logistic regression model including age group, oestrogen receptor (ER) status and year of surgery in addition to those listed above.
